# Like or Dislike? Affective Preference Modulates Neural Response to Others' Gains and Losses

**DOI:** 10.1371/journal.pone.0105694

**Published:** 2014-08-29

**Authors:** Yang Wang, Chen Qu, Qiuling Luo, Lulu Qu, Xuebing Li

**Affiliations:** 1 School of Psychology, Center for the Study of Applied Psychology, South China Normal University, Guangzhou, China; 2 Department of Psychology, The Chinese University of Hong Kong, Hong Kong, S.A.R., China; 3 Key Laboratory of Mental Health, Institute of Psychology, Chinese Academy of Sciences, Beijing, China; University of Tuebingen Medical School, Germany

## Abstract

Previous studies have demonstrated that the brain responds differentially to others' gains and losses relative to one's own, moderated by social context factors such as competition and interpersonal relationships. In the current study, we tested the hypothesis that the neural response to others' outcomes could be modulated by a short-term induced affective preference. We engaged 17 men and 18 women in a social-exchange game, in which two confederates played fairly or unfairly. Both men and women rated the fair player as likable and the unfair players as unlikable. Afterwards, ERPs were recorded while participants observed each confederates playing a gambling game individually. This study examines feedback related negativity (FRN), an ERP component sensitive to negative feedback. ANOVA showed a significant interaction in which females but not males displayed stronger FRNs when observing likable players' outcomes compared to unlikable ones'. However, males did not respond differently under either circumstance. These findings suggest that, at least in females, the neural response is influenced by a short-term induced affective preference.

## Introduction

Although committing an error or receiving a negative feedback is generally considered to be an unpleasant event, it is crucial for learning and for adjusting future behavior. Converging evidence implies that FRN (feedback-related negativity), an ERP component generated from the anterior cingulate cortex (ACC), is generally more pronounced for negative outcomes of our own performances. The FRN peaks approximately 200-300 ms after feedback onset, and is related to the learning of information that can guide subsequent behaviors [1]–[4]. As social creatures, humans learn not only from their own experiences but also by observing others' behaviors during social interactions [5]. A growing number of studies have demonstrated that observing another's monetary loss elicits an FRN effect that is similar, both in morphology and scalp distribution, to a loss that is directly experienced, a pattern commonly referred to as oFRN (observational-FRN)[5]–[7]. This phenomenon implies that the neural process underlying “learning by observation” resembles the process underlying “learning by doing”. This similarity allows us to avoid negative results that are potentially dangerous without having to experience them directly.

By manipulating the relationship between an observer and another person, previous studies suggested that the oFRN might be driven by two processes. First, one may evaluate the observer's outcome from an egocentric perspective when one's own benefit is involved. For example, the research in which an observer and a performer were in competition so that the observer lost when the performer won and vice versa, showed that the oFRN in response to wins of the performer was similar to that in response to losses of the observer[8], [9]. Even participating in one's own separate gambling game alongside the observational task could affect feelings about others. Leng and Zhou (2010) explored the differing neural responses to friends and strangers when the observer was engaged in the same gambling game, and failed to find a differentiation of FRN responses between friend and stranger observations This finding was interpreted as shown that participation itself might draw their attention from other's performance to their own benefit, potentially diminishing the expected differences in neural response toward friends versus strangers[10].

Second, an observer may evaluate a performer's outcome empathically and emotionally when it has no consequence for the self. Modulation of the oFRN occurs when the observer's outcomes are unrelated to the performer's, and the existence of a long-term relationship has been found to modulate the magnitude of oFRN when self-involvement is not a factor in the experiment. Ma and colleagues removed the observer's own gambling from the experimental design, so that participants only observed and evaluated the outcomes of friends versus strangers. Once the observers' egocentric focus on their own results was no longer a factor, there was a larger oFRN effect associated with friends' outcomes than with strangers' [11]. It seems like that the greater the self-benefit is involved, the more that egocentrism and cognitive evaluation reduce the emotional response toward others' results. The less self-benefit is involved, the more emotion influences the oFRN.

However, the design that does not include participation in the same gambling game cannot exclude personal involvement as an influence on oFRN. It also could be argued that it is the potential benefits gained from the success of the other person in a long-term relationship that results in the differentiation seen in the oFRN phase. For example, a friend might pay for drinks after winning a game. Therefore,it is unclear whether the stronger response to friends' losses and gains is due to the social context or to this potential personal benefits.

Another possible interpretation of the larger oFRN in response to friends' outcomes is that one may generally attend more to friends than to strangers because of higher familiarity. The purpose of the present study was to investigate the degree to which the oFRN is moderated by affective preference when controlling for potential benefit and familiarity. A variation on the trust game was used to establish a short-term affective preference for two strangers [12]. In the game, one confederate was fair and generous, while the other was unfair and selfish, leading to varying affective preferences on the part of the participant. Then, the two confederates played a gambling game individually while the participant observed. Event-related potentials (ERPs) were recorded during the observation phase. This paradigm allowed us to explore how a learned affective preference influences neural responses toward liked and disliked strangers. It was expected that the learned affective preference would result in a larger oFRN toward the likable player than toward the unlikable one.

Gender differences were also examined in the current study. Although females are better at recognizing emotions and at expressing themselves emotionally, males show greater responses when presented with threatening emotions such as fear, anger and dominance (see a review, [13]). For example, females tend to be sensitive to both positive (happy) and negative (angry) emotions, but males are primarily sensitive to the negative one (i.e., angry) [14]. This pattern might be due to sex differences in hormones, chromosomes, and brain structures [13]. Besides gender differences in the emotion expression and recognition, females often have a stronger empathic responses than males and score higher on self-reported empathy [15]. When accessing others' emotions females showed increased activation in the right inferior frontal cortex while there is no differential activations in males. Females also recruit areas of the human mirror system, which has been claimed to be related to the capacity for empathy, to a higher degree than males during both self assessing and other assessing tasks [16]. Furthermore, from an evolutionary perspective, males might benefit more than females from competition. For example, the copulation frequency of male elephant seals during breeding seasons is related directly to success in male-male competition. Low competitive males that reached maturity are prevented from mating by the highest ranking males[17]. There is also evidence that in humans, an intergroup conflict has profound effects on males in particular compared with females [18], [19].

Considering the fact that females showed stronger emotion sensitivity and empathy responses compared to males, it might be easier for them to distinguish the results of fair players and unfair players, whereas males might be especially more sensitive to unfair players only, and might even use an egocentric evaluation path to process unfair players' results, and present a reversed oFRN pattern. This hypothesis is further supported by Singer and colleges' findings that women showed more empathy toward fair versus unfair players' pain, whereas men's reward region was activated even when they faced unfair players' losses[12]. Thus, we predict that compared to women, men would show stronger sensitivity to unfair players' results.

## Methods

The study was approved by the Ethics Committee of the department of Psychology at South China Normal University. Written informed consents were obtained from participants, and they had the right to discontinue participation at any time.

Forty participants (20 male and 20 female) aged 19–25 years (Mean  =  22 years, SD  =  2.1 years) were recruited from South China Normal University, China. Five participants (three male and two female) were excluded from ERP data analysis, one because one fell asleep during the experiment and the other four because they correctly identified the other two “participants” as confederates. All participants had normal or corrected-to-normal vision and no history of neurobiological or psychiatric disorders. Participants were paid 30 yuan (about $4.50) for their participation.

### Procedure

Participants sat in an electrically shielded room about 1 m away from and in front of a 17-inch CRT while their EEG was recorded simultaneously. They were told that two other students were sitting in different rooms and would play games with them. There would be three separate games: one was related to “social exchange”, one was an “observational-learning task”, and one was related to “grouping-social exchange” in which participants would be able to invite one co-player to form a team to compete against the other team. Participants did not actually play the third game which was mentioned only to assure participants that they were playing with real people rather than a computer program.

**Figure 1:**
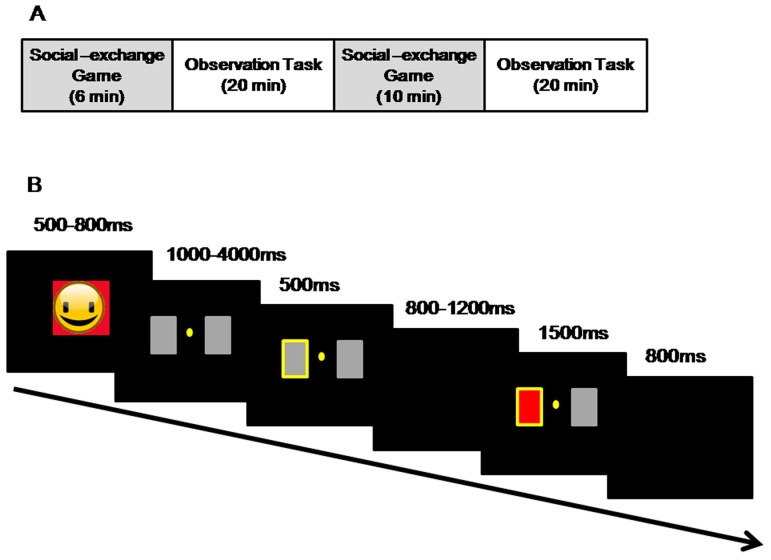
(A) Schematic representation of the experimental procedure. The study included two independent experiments. One experiment was a social exchange game and the other one was an observation task. Each experiment consisted of two blocks which were alternated, and the study ended with a behavioral assessment. (B) Observation task: overview. (The vectogram for the human face used here is provided for illustration, photographs of human faces were used in the study.)

Each of the two games was divided into two sections. The experiment started with one section of the social exchange game, followed by two sections of the observational learning task which was interspersed by a second section of the social exchange game. At the end of the experiment, participants completed a behavioral assessment, see Fig. 1(A). They were then fully debriefed about the aims and methods of the study. Participation in the entire study took around one hour and thirty minutes.

### Social-exchange game

We used a revised trust game, which has also been adopted in previous studies to induce participants' feeling of like and dislike towards strangers[12], [20]. In the traditional trust game, subject A could choose any amount of money from zero to ten dollars, to send to subject B. The amount of money would be tripled by the time it reached subject B. Subject B would then decide how much of the tripled money to keep and how much to send back to the sender [20]. In the current study, each experimental group was composed of one participant and two same-gender strangers. The participant always had the first move and was given the choice of “trusting” the confederate by sending 10 starting points or “mistrusting” by keeping the points. According to the game rules, points sent would be tripled. A confederate who received these 30 points reciprocated by sending between 0 and 10 points back, which were also tripled. The fair player reciprocated with large amounts, whereas the unfair player reciprocated with small amounts.

Photos of the two confederates and the participant, taken before the experiment, were used in a computer program to remind participants whose turn it was and to make the setup more convincing. The photos had backgrounds with different colors: blue for participants, red for one confederate, and green for the other (counterbalanced across participants). The same colors were displayed on playing cards in the observation task so that participants could easily distinguish between likable and unlikable confederates easily.

### Observational-learning task

Following the exchange game, participants were informed that the two participants they played with would then play a gambling game individually in which they would win money or lose money from their original payment. To maintain their attention, participants were asked to count the number of losses or gains in each block.

The observation task consisted of 240 trials: 120 games played by the unfair confederate and 120 by the fair confederate. The 240 trials were split into 12 blocks of 20 trials, each performed by one confederate. The gambling task was adapted from Gehring and Willoughby's (2002) classic task, illustrated in Fig. 1(B). In the original task, the participant was asked to choose from two squares, each of which contained the number 5 or 25. After the choice, each square turned red or green indicating whether the participant lost or won such amount of money[21]. In the current study, each trial began with the participant's photo (3.5° × 5°) against a black background for 500–800 ms. Two gray squares then appeared, laid out horizontally, for 1000–4000 ms. Each subtended 1.6°x 1.6°, and the visual angle between the centers of the two squares was 3 degrees. The player whose turn it was selected one card with a key press, pressing the ‘F’ key for the left card or ‘J’ for the right card. The chosen card was then highlighted with a yellow border for 500 ms. After another 800–1200 ms interval, the background of the performer's chosen card turned the color displayed in his/her social-exchange game, with a ‘+’ or ‘−’ on it to show whether he/she gained or lost on that trial. The inter-stimulus interval was 800 ms.

### Electrophysiological recordings

EEGs were recorded from 32 scalp sites using tin electrodes mounted in an elastic cap (Brainproducts, Munich, Germany) with the reference on the left mastoid. Eye blinks were monitored with electrodes located in four places: above and below the right eye and 1.5 cm lateral to the left and right external canthi. All electrode recordings were referenced to an electrode placed on the left mastoid, and electrode impedances were kept below 5 k Ohm for all recordings. Off-line analysis was performed using Brain Vision Analyzer software (Brainproducts).The electrophysiological signals were filtered with a bandpass of 0.01–100 Hz and digitized at a rate of 500 Hz. Trials with amplitudes of more than ± 100 uV in EEG voltages were excluded from further analysis. EEG data were digitally filtered below 30 Hz (24 dB/Octave) and re-referenced offline to linked mastoid electrodes. ERPs time locked to feedback (gains and losses) were averaged for epochs of 1000 ms, using a 200 ms pre-feedback baseline.

The ERP components analyzed in this study include the FRN and P300. For the purpose of statistical analysis, we selected two electrodes, Fz and FCz, in the anterior frontal midline area for FRN, because the FRN effect was largest at these electrodes, and they had been commonly found to produce large FRN effects in previous studies[22]–[25].

A 2 (gain vs. loss) by 2 (likable vs. unlikable) by 2 (female vs. male) repeated-measures ANOVA on the peak latencies of FRN found only a significant gender difference, *F*(1, 33)  =  9.099, *p*  =  .005, *η2* = .216, in which males displayed a shorter peak FRN latency (266.89 ±7.70 ms) than did females (299.28 ± 7.49 ms). Other main and interaction effects were all non-significant (*p*>.05). Based on this, we chose a different time window for FRN in analyses on females and males. FRN was defined as the mean amplitude of the outcome distributed on the anterior scalp at 240–340 ms for females and at 200–300 ms for males.

## Results

### Rating-task results

To check whether the affective-preference manipulation worked, participants performed a likeability trait rating task at the end of the experiment using a 7-point Likert scale ranging from -3 (unlikable) to 3 (ikable). There was a significant difference between perceptions of the two strangers (*F*(1, 33)  =  194.15, *p* < .01, η^2^ = .885), indicating a preference for the fair player (Mean = 2.51, *SD* =  0.13) over the unfair one (Mean  =  −1.14, *SD* =  1.22). No significant gender difference was found (*F* < 1).

### ERP results

**Figure 2:**
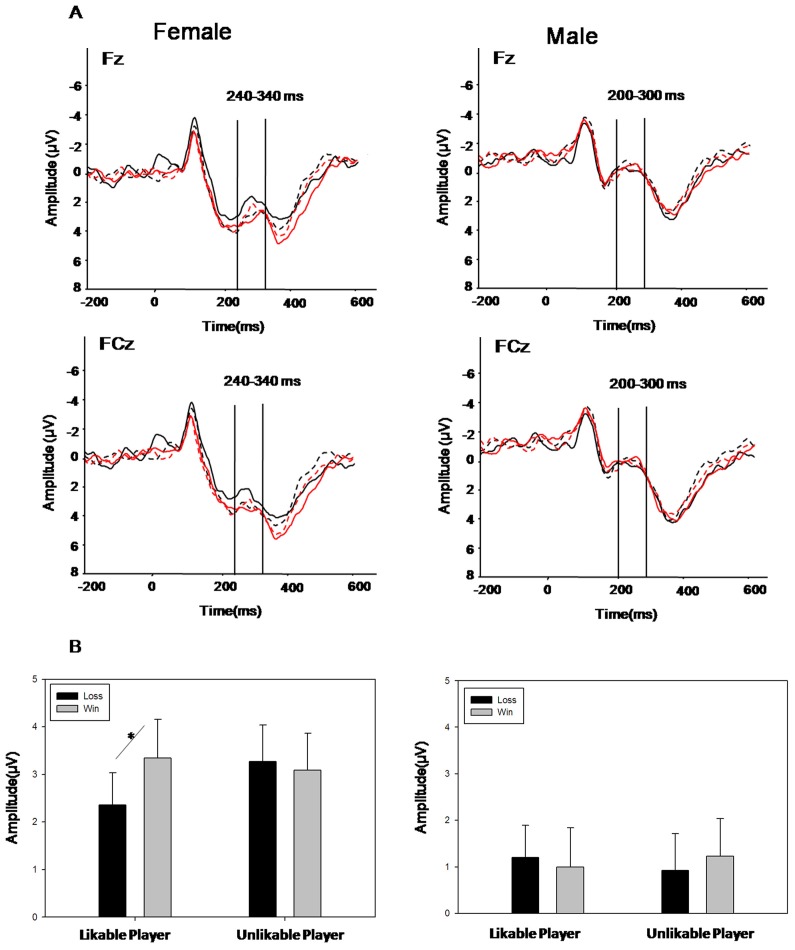
The ERP grand-average waveforms and the amplitudes of the FRN. (A) The grand-averaged ERP waveforms aligned at the onset of feedback stimuli for likable and unlikable players' performance (recorded for Fz and FCz sites). The left panel shows data from females, and the right panel shows data for males. (B) FRN response to win and loss outcomes of likable and unlikable players based on gender.

Fig. 2(A) shows the event-related potentials for gains and losses at Fz, based on likeability and gender. The analysis of mean FRN amplitudes with four factors (agency, valence, gender, electrode) revealed only one significant interaction effect a three-way interaction among agency, valence and gender (*F*(1, 33)  =  4.60, *p*  =  0.039, *η^2^* = .12), indicating that the FRN was influenced by both affective preference and gender. No other significant main effect or interaction effects were found. Follow-up simple-effects analysis showed that,for women,the main effect of valence was significant when observing likable players' outcomes (*F*(1, 33)  =  4.42, *p*  =  .043, *η^2^* = .535),whereas the comparison between unlikable players' loses and gains was not significant ( *F*(1, 33)  =  0.37, *p*  =  .574). Mean ERP amplitudes were significantly more negative in response to the likable players' losses (2.354 ± 0.677 µV) than to their wins (3.27 ± 0.768 µV) in females (*F*(1,33) = 4.42, *p* = 0.043). Female participants also showed more negative mean ERP amplitudes in response to the likable players' losses (2.354 ± 0.677 µV) than to those of the unlikable player (3.343 ±0.813 µV) (*F*(1, 33)  =  5.92, *p*  =  0.021, *η^2^* = .551, See Fig. 2(B). However, for men, the main effect of valence was not significant when neither observing likable players' performance (p = .538) nor unlikable players' performance (p = .592).

## Discussion

Previous researches on the FRN effect under observation conditions have focused on manipulating the relationship between self and others by taking advantage of long-term friendships or by using a benefit-related competition situation. The present study, using trust game and gambling game, extends this work by examining the gender difference in how the short-term social interaction induces affective preference modulation in oFRN. The results suggest a gender-based difference in rapidly processing others' losses and gains. For females, the likable performers' losses elicited larger oFRN than did those of unlikable performers. Although males rated the fair player as more likable, they did not display significantly different responses toward the two performers. The results imply an existence of emotional and empathic explanation of oFRN.

The female having generally sensitive neural response for distinguishing likable from unlikable strangers is concordant with previous studies and implies an empathetic and emotional account of oFRN. It has been reported that female participants categorized a friend's loss as equivalently negative to their own even in a competitive situation[26]. The dual-processes hypothesis suggests that the other person's status as a personal acquaintance might have enhanced females' empathic responses, overriding the egocentric evaluative component for female participants[9]. In addition, empathy might be the cause of observational FRN[9]-[11], [26]. In the previous studies, the size of the FRN effect varied as a function of whether an action was performed by a friend or by a stranger[11]. In another study, females showed a smaller empathic response toward unfair players' pain[12]. In the present study, the greater FRN responses for the likable player in the observation condition, similar to responses induced by feedback information concerning the observer's own results, under the observation condition may be related to a stronger empathic response toward the likable player. When observing the unlikable players' results, the observer's reduced empathic response might result in a non-significant FRN effect. These results might also be supported by Wilson's selection theory in which the units of evolution is group instead of individual, and highly cooperative groups have an evolutionary advantage over poorly cooperative groups [27]. People tend to cooperate with in-group members whereas they compete with out-group members to benefit themselves. From this perspective, the human tribal inclination has evolved to help humans categorize individuals based on their group membership, and treat in-group members benevolently and out-group members malevolently[19]. The likable player in our study might be considered as an in-group member while the unlikable player might be treated as an out group member. Thus, we observed a disassociation between the responses to these two members.

Crucially, the current study revealed a gender effect in which females responded differently toward likable versus unlikable performers, whereas males did not. This inconsistent response toward two strangers suggests that, in females, not only long-term relationships but also the affective link established through short-term social interaction can affect early neural responses to the consequences of actions unrelated to one's own interests. We suggest that this phenomenan is caused by different empathic responses in females from that in males. The gender-based differences in empathy have been widely reported. Using self-report questionnaires, females showed superiority in empathy in the general population and even among persons with Asperger Syndrome(AS)/high-functioning autism(HFA) [15]. Using voxel-based morphometry analysis, concurrent with the dispositional empathy measures, researchers also found that young adult females had significantly larger gray matter volume locatged in the mirror-neuron system, which is highly related in empathy ability[28]. Females showed earlier and stronger brain processing of the action's purpose in the females brain compared with males in an ERP study[29]. Females were also more accurate when their feelings of the target[30].

Gender-based differences in emotion sensitivity and interpersonal sensitivity might also be a reason for the gender differences were found in the current study. Females often perform better in various emotional tasks than males. Studies focused on sex differences in facial expression processing found that female advantage in decoding of emotional cues both in adults and children[31].Consistently, females displayed superior performance in affective arousal and expression of emotion over males. For instance, increased corrugator activity for angry faces and increased zygomatic activity for happy faces were more pronounced for females compared to males[32]. Unpleasant and high arousing stimuli also evoked stronger N100 and N200 in females compared to males[33].

In addition, Singer and her coworkers found that males expressed a desire for revenge when observing players who had just betrayed them by administering pain[12]. However, males' hypothesized reversed responses toward unfair player did not occur in our study. It is possible that males did not process the information. This explanation might be supported by the shorter latency of oFRN in males compared with that in females. Females' automatic response toward others may lead to a longer latency in which to make sure that they could separate the likable player and unlikable one, whereas the males, who finished the evaluation phrase in a shorter time, could not finish an precise evaluation. Males' weaker empathic/emotional response and lower level of social sensitivity might not allow them to separate their representations of the two strangers within a few hundred million seconds. Finally, from an evolutionary perspective, males should show stronger sensitivity to unfair players' results, but our results were not completely in line with the evolutionary hypothesis. However, the rapid evaluation failure does not mean it will not work in the long run. Using more extreme targets (people one hates), the revenge effect on oFRN might be possible.

By manipulating observers' affective preferences toward different performers through trust games and by removing the effect of self-benefit, the present study found that females displayed a stronger FRN effect when observing likable players' outcomes compared to unlikable players' and a stronger P300 response toward unlikable ones; males did not display any difference in their responses toward the two strangers. These findings suggest that, at least in females, outcome evaluation can be affected by short-term affective preference even when the person being observed is a total stranger.
