# Correction: Like or Dislike? Affective Preference Modulates Neural Response to Others' Gains and Losses

**DOI:** 10.1371/journal.pone.0114200

**Published:** 2014-11-18

**Authors:** The PLOS ONE Staff

The affiliation for the second author is incorrect. Chen Qu is not affiliated with #2 but with #1 School of Psychology, Center for the Study of Applied Psychology, South China Normal University, Guangzhou, China.

## References

[1] Wang Y, Qu C, Luo Q, Qu L, Li X (2014) Like or Dislike? Affective Preference Modulates Neural Response to Others' Gains and Losses. PLoS ONE 9(8): e105694 doi:10.1371/journal.pone.0105694 25171075 10.1371/journal.pone.0105694 PMC4149476

